# Blood biomarkers of hepatocellular carcinoma: a critical review

**DOI:** 10.3389/fcell.2024.1489836

**Published:** 2024-11-22

**Authors:** Junsheng Zhao, Zekai Hu, Xiaoping Zheng, Yajie Lin, Xiao Liu, Junjie Zhang, Jing Peng, Hainv Gao

**Affiliations:** ^1^ Key Laboratory of Artificial Organs and Computational Medicine in Zhejiang Province, Shulan International Medical College, Zhejiang Shuren University, Hangzhou, China; ^2^ State Key Laboratory for Diagnosis and Treatment of Infectious Diseases, National Clinical Research Center for Infectious Diseases, National Medical Center for Infectious Diseases, Collaborative Innovation Center for Diagnosis and Treatment of Infectious Diseases, The First Affiliated Hospital, Zhejiang University School of Medicine, Hangzhou, China; ^3^ Hangzhou Tongchuang Medical Laboratory, Department of pathology, Hangzhou, China; ^4^ Key Laboratory of Artificial Organs and Computational Medicine in Zhejiang Province, Shulan (Hangzhou) Hospital Affiliated to Shulan International Medical College, Zhejiang Shuren University, Hangzhou, China; ^5^ Department of Breast Surgery, Renji Hospital, School of Medicine, Shanghai Jiao Tong University, Shanghai, China

**Keywords:** hepatocellular carcinoma, early diagnosis, blood biomarkers, biomarker detection technology, prognosis

## Abstract

Hepatocellular Carcinoma (HCC) is a malignant tumor with high morbidity and mortality worldwide, which represents a serious threat to human life, health and quality of life. Blood-based detection is essential for HCC screening, early diagnosis, prognosis evaluation, and surveillance. Current non-invasive detection strategy including serum alpha-fetoprotein (AFP), ultrasound, computerized tomography, and magnetic resonance imaging. The limited specificity of an AFP and the dependence on operator experience and diagnostic personnel for ultrasound have constrained their utility in early HCC diagnosis. In recent years, with the development of various detection technologies, there has been an increasing focus on exploring blood-based detection markers for HCC. The types of markers include protein markers, DNA mutation, DNA epigenetic modification, mRNA, miRNA, and so on. However, numerous methodological and biological factors limit the clinical sensitivity and generalization performance of these new biomarkers. In this review, we describe the state-of-the-art technologies for cfDNA analysis, and discuss outstanding biological and technical challenges that, if addressed, would substantially improve HCC diagnostics and patient care.

## 1 Introduction

Liver cancer is one of the most common cancers, the sixth most common cancer in the world, with >860,000 new cases and >750,000 deaths in 2022 (Bray et al., 2024), and the fifth most common cancer in China (Zhang et al., 2021). Primary liver cancers include hepatocellular carcinoma (HCC) (75%–85% of cases) and intrahepatic cholangiocarcinoma (10%–15% of cases), as well as other rare types. The onset of liver cancer is occult, lacking typical clinical symptoms in the early stage, and many patients are already in the advanced stage when they are found, losing the opportunity for surgery. However, systemic chemotherapy or radiotherapy is not ideal for primary liver cancer, immune-checkpoint inhibitor (ICI)-based therapies only ∼30% of advanced-stage HCC patients have an objective response, and liver cancer has become the third highest mortality cancer. In most countries, liver cancer survival rates have barely improved from 1995 to 2022, and the 5-year survival rate remains far below 20% (Villanueva, 2019). Even in the United States, where medical conditions are better, liver cancer death rates have been rising while mortality rates for other types of cancer have declined (Bray et al., 2018). More than half of the world’s liver cancer cases and deaths occur in China. In recent years, data show that the incidence and mortality of liver cancer are on the rise globally, and the incidence of men is twice that of women (Rumgay et al., 2022).

## 2 Risk factors for HCC

The main risk factors for liver cancer are chronic infection with hepatitis B virus (HBV) or hepatitis C virus (HCV), aflatoxin-contaminated food, heavy alcohol consumption, obesity, smoking and type 2 diabetes. Major risk factors vary by region. In most high-risk HCC regions (China, East Africa), the key risks are chronic HBV infection and aflatoxin exposure, while in other countries (Japan, Egypt, etc.), HCV infection may be the main cause. In Mongolia, HBV and HCV and co-infection of HBV carriers with HCV or hepatitis E virus (HEV), as well as alcohol abuse, contribute to the high incidence of liver cancer. The increase in obesity is also responsible for the increased incidence of low-risk liver cancer. Studies have found that diabetes can increase the risk of cancer in hepatitis B patients by 2 to 3 folds. Patients with a glycated hemoglobin level of more than 9% and chronic liver disease (alcoholic liver injury, cirrhosis, HBV or HCV infection, and other chronic liver disease) have a higher risk of liver cancer. In addition, diabetes can significantly reduce the survival rate and increase the recurrence rate of liver cancer (Alexander et al., 2019; Pang et al., 2018). In addition to control of HBV infection, HBV vaccination might avert the development of liver cancer and reduce the risk of death due to liver cancer. Therefore, HBV vaccination was recommended as primary prevention of liver cancer since 1982. WHO recommends its incorporation into routine infant immunization programme and by the end of 2016, 186 countries had introduced HBV vaccine into heir national immunization schedules. The vaccine has dramatically reduced the prevalence of HBV infection and the incidence of HCC in young people in high-risk countries where mass vaccination is being introduced. However, there is currently no vaccine to prevent HCV infection. Although HCV transmission has declined substantially in resource-rich countries, the continued use of contaminated needles and unsafe blood transfusions in some low-income countries are also important contributors to the spread of infection. Recent advances in anti-HBV and HCV therapy suggest that, although currently expensive, HCC risk can be significantly reduced (Kanwal et al., 2017; Yang et al., 2022).

Metabolic fatty liver disease (MFLD) not only increases the risk of HCC but also HCC-related mortality, but it does not increase the risk of HCC recurrence or all-cause mortality, suggesting the need for interventions in the MFLD population and for HCC surveillance in patients with metabolic steatohepatitis and fibrosis (Cholankeril and Kanwal, 2021; Norero and Dufour, 2023; Orci et al., 2022; Thomas et al., 2024).

Understanding these risk factors is helpful for risk stratification of the population to improve the precision of screening and save medical resources.

## 3 Implications of blood-based biomarker

In recent years, tremendous progress has been made in the treatment of HCC. However, all treatment options are only feasible if diagnosed early. According to the 2019 Cancer Report of the United States of America, the key to reducing cancer mortality is early detection and treatment of cancer, which has almost become common knowledge in the field of cancer (Siegel et al., 2019). HCC surveillance is of high value in high-risk individuals, for example, hepatitis virus carriers, patients with cirrhosis (Singal et al., 2022). However, the insidious onset and atypical symptoms of liver cancer make it difficult to detect early liver cancer. Finding reliable biomarkers for early diagnosis of liver cancer has become a key way for early detection of liver cancer (Parikh et al., 2020). Early HCC screening/diagnostic biomarkers should have the following characteristics (Kimhofer et al., 2015):

1) The target marker should be detectable in noninvasively obtained samples such as blood or urine;
2) The marker should have good diagnostic and/or prognostic performance (e.g., high sensitivity and specificity);
3) The markers should be able to be tested with reliable and stable equipment, and the test results should be interpreted in the field according to a simple format without additional equipment; This standard essentially excludes Next-generation sequencing (NGS) technology;
4) Testing should be inexpensive so that it is available to all who need it;
5) Biomarkers should be validated in a wide range of populations.

Additionally, we believe that prospective studies should be conducted in populations with hepatitis virus infection to verify the performance of markers for early screening of HCC before conducting applicability studies in all populations (Sachar et al., 2021).

## 4 Current guidelines and consensus

Early diagnosis of liver cancer is of great significance, and domestic and foreign guidelines emphasize early screening and detection of liver cancer. Serum alpha-fetoprotein (AFP) and liver ultrasound are the two most commonly used methods for liver cancer screening. The main serological biomarker of liver cancer is serum AFP, which is the most commonly used diagnostic method at present. The positive diagnostic cut-off value of serum AFP is >400 ng/mL. It is worth noting that the main application of AFP detection is in diagnosis, rather than screening and surveillance. The test typically has a sensitivity of only about 40%–60%, a rate of false-negative of 30%–40%, a specificity of 80%–90% (Huang et al., 2022; 2023; Yang et al., 2019). AFP can also be elevated in chronic hepatitis, liver cirrhosis and other diseases, which can easily lead to false positive results when used for tumor screening. This is also an important reason why the American Association for the Study of Liver Diseases (AASLD) guidelines and the European Association for the Study of the Liver and the European Organization for Research and Treatment of Cancer no longer use AFP as a screening index (Bruix et al., 2011; Galle et al., 2018; Singal et al., 2023). However, AFP serological test is still widely used as a screening index for HCC in the Asia-Pacific region. The Asian-Pacific Association for the Study of the Liver (APASL) recommends surveillance for HBV-infected men older than 40 and women older than 50: Liver ultrasound and AFP detection should be performed every 6 months, and areas where conditions are available can consider increasing the screening frequency or other tumor markers and imaging examinations, such as DCP, AFP-L3, CT/MRI, etc. (Omata et al., 2010). In addition, Tong et al. (2010) suggested that surveillance for liver cancer can improve the survival of patients with hepatitis B and should be included in the standard medical regimen for patients.

The European Association for the Study of the Liver and the European Organization for Research and Treatment of Cancer jointly recommend the detection of heat shock protein (HSP70), glypican (GPC3) and glutamine synthetase (GS) to improve the early diagnosis rate of HCC (Galle et al., 2018). The International Consensus Group of Hepatocellular Neoplasia and the World Health Organization (WHO) has also included these three tests in their recommendations (Nagtegaal et al., 2020).

In summary, the main problems of the currently widely used liver cancer screening programs are insufficient sensitivity, low accuracy, and poor compliance due to invasiveness (Zeng et al., 2023). There is an urgent need to develop more accurate and less invasive detection methods that can detect smaller tumor size and be suitable for general population screening. In theory, detection of circulating tumor cells (CTC), circulating cell-free DNA (ccfDNA), circulating RNA (mRNA, miRNA, lncRNA), circulating proteins or exosomes are potential target markers (Parikh et al., 2023).

## 5 Blood-based biomarkers of liver cancer

**FIGURE 1:**
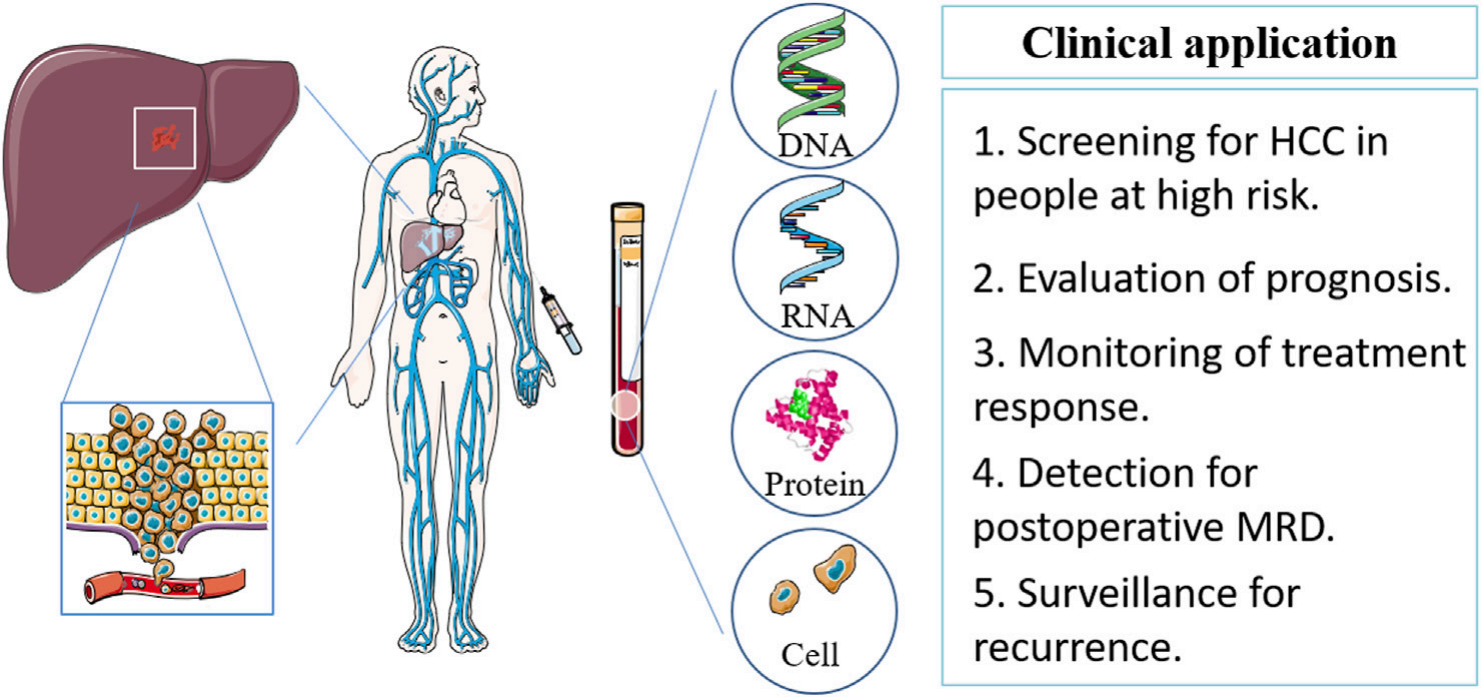
Blood-based biomarker sources, types and clinical implications in HCC. HCC, hepatocellular carcinoma; MRD, minimal residual disease.

In addition to traditional biomarkers, AFP, newer blood-based biomarkers such as ctDNA, microRNAs, circulating tumor cells, and exosomes are being investigated for their utility in HCC diagnosis and prognosis. These biomarkers offer the potential for more accurate and sensitive detection of HCC, as well as monitoring of treatment response and disease progression (Figure 1).

### 5.1 Protein markers

#### 5.1.1 Serum alpha-fetoprotein (AFP)

Serum protein markers is most current clinical practice of blood markers for HCC, and new protein markers are emerging due to the development of proteomics technology (Table 1). The most widely used HCC biomarker is AFP that sensitivity for HCC range from 40% to 65%. Semiannual AFP screening of HBV carriers can lead to earlier diagnosis of associated HCC, but has no effect on five-year survival rate (Chen et al., 2003). Randomized controlled trials have shown that screening HBV carriers and patients with chronic hepatitis with AFP and ultrasound twice a year can reduce HCC-related mortality. Compared with the control group, the detection rate of HCC in the early and operable stages was significantly improved in the screening group (Zhang et al., 2004). Three kinds of AFP with different glycosyl modifications have been identified, among which AFP-L3 was more specific than AFP. Although the sensitivity of AFP-L3 detection is controversial, Oda et al. (2011) confirmed the clinical application value of AFP-L3 detection using a highly sensitive automatic immune system (Kagebayashi et al., 2009).

**TABLE 1:** Blood-based protein biomarker for HCC.

Test name	Clinical trial ID	Markers	Sample size	Advantages	Limitation	Clinical application	Reference
P4 panel	NCT03588442	HABP2, CD163, AFP, and PIVKA-II	HCC (*n* = 373), Liver cirrhosis (n = 421), basic liver diseases (n = 64), HBV carrier (n = 40), and healthy controls (n = 104)	Includes prospective study;	The equipment of PRM-MS is expensive and complex to operate, so it has not been widely used in clinical applications. Other less expensive serum protein detection techniques should be determine whether they are suitable for protein marker detection in this study.	Auxiliary diagnosis	Xing et al. (2023)
——	No registered	AKR1B10 and CTSA	HCC (*n* = 16), Liver cirrhosis (*n* = 24), and healthy controls (*n* = 24)	ELISA is fast and cheap, so it is suitable for tumor screening;	Sample size is small;	Auxiliary diagnosis	Du et al. (2020)
AFP	NCT02715531 NCT03434379	AFP	Patients with HCC (n = 150)	A simple assay might have important clinical applications	The sensitivity of AFP was not high.	Auxiliary diagnosis	Zhu et al. (2022), Kuzuya et al. (2022)
——	NCT03047603	PIVKA-II and AFP	Control (n = 1731) and patients with HCC (n = 1,194).	A web-based calculator including age, sex, AFP, and PIVKA-II. The tests are easy and cost is low.	Further studies of the sensitivity of this assay in early patient cohorts is needed. Prospective cohort studies in HBV-carriers are more convincing.	Auxiliary diagnosis	Yang et al. (2019)
——	——	IgM-free AIM	Control (n = 117) and patients with HCC (n = 95).	Serum IgM-free AIM may represent a universal HCC diagnostic marker superior to AFP or DCP.	Simpler alternatives than electrochemiluminescence immunoassay (ECLIA) could be investigated. The diagnostic performance of fAIM should also be explored in populations at high risk for HCC other than NAFLD.	Auxiliary diagnosis	Shimizu et al. (2022)
——	——	IgM-free AIM	Control (n = 175) patients with HCC (n = 42)	This longitudinal study explored the value of fAIM for the early diagnosis of HCC.	The sample size is small, and only include NASH patients. The clinical value in other types of high-risk populations, such as HBV carriers, should be further studied too.	Auxiliary diagnosis	Okanoue et al. (2022)
——	——	CHI3L1	Healthy control (n = 40); patients with HCC (n = 128), LC (n = 40), and chronic hepatitis (n = 40)	Serum CHI3L1 can help to increase diagnostic power and is an independent prognostic factor in patients with HCC.	The sample size was small.	Auxiliary diagnosis	Wang et al. (2022)
——	——	CCL20 and LCN2	Patients with HCC (n = 167), liver cirrhosis (n = 106), and healthy control (n = 106).	A combination model composed of CCL20 and LCN2 may serve as a more efficient tool than AFP for distinguishing HCC; ELASA is an common and easy to master method.	The sample size of early-stage HCC was small.	Auxiliary diagnosis	Du et al. (2022)
——	——	OPNI	Patients with HCC (n = 409) and benign liver disease (n = 345)	OPNI can be included as appropriate to improve the accuracy of monitoring recurrence of HCC.	Application in the early diagnosis value need further research.	Auxiliary diagnosis	Zhou and Yang (2023)
——	——	GAPR1, PLTP, CLASP2, IGHV1-69D, IGLV5-45, A2M, VNN1, KLK11, ANPEP, DPP4 and HYI.	HIV/HBV co-infection patients with HCC (n = 13) and non-HCC (125).	Proteomics was used to study the serum protein alterations in HIV/HBV patients with or without HCC.	The sample size is small and too many markers.	Auxiliary diagnosis	Ke et al. (2023)
——	——	OMA1 and YME1L	none	The first time confirmed that OMA1 is downregulated and YME1L is upregulated in HCC.	This study USES up animal model of rats, need further clinical study to illustrate the diagnostic value of markers.	Auxiliary diagnosis	Abass et al. (2023)
——	——	Alpha-1 antitrypsin (A1AT)	Healthy control (n = 100), patients with HCC (n = 100)	Blood A1AT concentration has a superior sensitivity than AFP measurement in detection of HCC.	Large sample size investigation need be carried out.	Auxiliary diagnosis	Sultan et al. (2023)
——	——	PIVKA-II and AFP	Cirrhotics with (n = 90) and without (n = 128) HCC.	The diagnostic accuracy of PIVKA-II was good in BCLC-B and excellent in BCLC-C patients.	Early liver cancer patient sample size is small.	Auxiliary diagnosis	Syriha et al. (2024)
——	NCT05719480	15 promising protein features.	Control (n = 27) and patients with HCC (n = 25)	Mass spectrometry (MS)-based proteomics analysis.	The sample size is small and too many markers.	Auxiliary diagnosis	Li et al. (2024)

#### 5.1.2 Serum prothrombin induced by vitamin K absence-Ⅱ (PIVKA-Ⅱ)

Several studies have found another useful protein biomarker is prothrombin induced by vitamin K absence-Ⅱ (PIVKA-Ⅱ), also known as Des-γ-carboxyprothrombin (DCP). It can promote the proliferation of HCC cells. The sensitivity of DCP for detecting HCC ranges from 48% to 62%, depending on the study and the stage of the cancer. Combined detection of other markers can further improve the sensitivity and specificity of early HCC diagnosis (Bae et al., 2011; Kamel et al., 2014; Poté et al., 2015; Xing et al., 2018).

#### 5.1.3 Serum glypican-3 (GPC3)

Studies have reported that the level of serum Glypican-3 (GPC3) in HCC patients is increased, which can accurately distinguish early HCC from cirrhosis, which secsitivity around 50%–72% (Hippo et al., 2004). Tangkijvanich et al. (2010) found that the serum GPC3 level was increased in HCC patients, which was not related to AFP. Combined detection of the two proteins can improve the sensitivity of early HCC detection. It has also been reported that combined detection of GPC3 and Heat shock protein 70 or heat shock protein 70 (HSP70) glutamine synthetase can more sensitively and specifically distinguish early or grade 1 HCC from cirrhosis (Silvia et al., 2012). These serum protein markers are generally cost-effective compared to other diagnostic tests. However, the conventional detection technology of protein sensitivity is not high, and protein biomarker lack of specificity.

#### 5.1.4 Others

New protein markers are still emerging. Some studies have also found that the Alpha-l-fucosidase (AFU) was elevated in serum of patients with cancer, included HCC (El-Houseini et al., 2005; Wang and Cao, 2004). That implicate its low specificity. DKK1 (dickkopf-1) protein was reported first time as a biomarker for HCC in 2012 (Shen et al., 2012). Its sensitivity of DKK1 protein for early HCC and small HCC was 70.9% and 58.5%, and the specificity was 90.5% and 84.7% (Shen et al., 2012). Liu Jie’s team from Fudan University obtained DKK1 aptamers with different dissociation rates in serum samples, and expected to replace DKK1 capture antibody of enzyme-linked immunoassay in early HCC detection (Zhou et al., 2019). Laminin (Ln)-332 contains three strands, α3, β3, and γ2. Recently, Japanese scientists have found that Ln-γ2/Ln-322 plays an important role in the occurrence and development of HCC, and Ln-γ2 monomer can be used as a potential biomarker to diagnose early HCC (Kiyokawa et al., 2017). This team evaluated the diagnostic value of Ln-γ2, AFP, and PIVKA-II in differentiating patients with chronic liver diseases (CLD) from HCC. The results showed that Ln-γ2 was more effective than AFP in differentiating CLD from HCC (Yasuda et al., 2019). In addition, such as Cytokeratin 19 (CK19), Golgi protein 73 (GP73), Osteopontin (OPN), heparin binding cytokines (Midkine, MDK) and Annexin A2 have also been shown to have the potential as early HCC diagnostic markers.

### 5.2 5-methylation-cyanine (5 mc) DNA biomarkers

Some new cfDNA markers we summarized in Table 2. Among them, aberrant 5 mc DNA methylation alterations, including hypermethylation of CpG islands (CGIs) and extensive hypomethylation, are a hallmark of HCC. DNA methylation is one of the most well-studied epigenetic modification mechanisms. It widely exists in the mammalian genome, and the level and pattern of genome methylation are specific in different tissues. DNA methylation detection technology has experienced comprehensive development in the era of high-throughput gene sequencing (Table 2).

**TABLE 2:** Selection of studies of cfDNA for detection of hepatocellular carcinoma.

Test name	Clinical trials ID	Biomarkers	Assay	Sample size (*n*)	Comments	Clinical application	Reference
——	——	*RNF135, LDHB*	MS-HRM	Healthy (*n* = 202), at-risk (*n* = 211), and patients with early HCC (*n* = 170) and Late HCC (*n* = 143)	The method is simple but has low sensitivity (70%).	Auxiliary diagnosis	Kim S. et al. (2023)
epiLiver	NCT03483922	cg02012576 (*CHFR*), cg03768777 (*VASH2*), cg05739190 (*CCNJ*), cg24804544 (*GRID2IP*), cg14126493 (*F12*)	Targeted bisulfite sequencing (NGS)	Healthy control (*n* = 96), patients with chronic hepatitis B (*n* = 51), non-HCC cancer (*n* = 102), and HCC (*n* = 302)	Only five methylation sites, can try to more simple detection method.	Auxiliary diagnosis	Cheishvili et al. (2023)
HepaAiQ	NCT05431621	twenty methylated regions	qMSP	Control (*n* = 735) and patients with HCC (*n* = 563).	The model was validated in a relatively large cohort. qMSP method is easy to learn, but is not suitable for detection of so many sites.	Auxiliary diagnosis	Guo et al. (2024)
CancerSEEK	——	CancerSEEK evaluates levels of eight proteins and the presence of mutations in 1933 distinct genomic positions.	target sequencing and	Healthy controls (n = 850) and patients with HCC (n = 39)	Can detect the presence of eight common solid tumor types. Prospective studies in a large population are required.	Auxiliary diagnosis	Cohen et al. (2018)
——	NCT05685524	*GNB4*, *Riplet*	qMSP	Healthy control (n = 98), patients with chronic liver disease (n = 199) and HCC (n = 173).	The method is simple and the sample size is small.	Auxiliary diagnosis	Zhao et al. (2024)
HcSeer	——	methylation-related features and fragmentomics features.	target sequencing	Healthy control (n = 480), patients with HCC (n = 342).	This study is available in the form of a conference abstract, and no study details have been made public.	Auxiliary diagnosis	Liu et al. (2023)
——	——	DNA methylation, fragmentation, and CAN landscape	whole-methylome sequencing (WMS)	Healthy control (*n* = 497), HCC (*n* = 113)	As a proof-of-concept study, there is still a lot of research to be done before the clinical application.	Auxiliary diagnosis	Bie et al. (2023)
——	——	fragmentation pattern	long-read sequencing	Normal (n = 25), patients with HCC (n = 48), and HBV carriers (n = 19).	Further studies are needed to characterize the specific long cfDNA molecular characteristics of HCC; Clinical application of long-read sequencing technologies need to establish a standard operating procedures.	Auxiliary diagnosis	Che et al. (2024)
PreCar Score	NCT03588442	CNV, genomic features (NF/5′ end motif/5 hm C/fragment size), and	WGS and 5 hm C sequencing	patients with LC (n = 4,367) and HCC (n = 510)	This work is a multi-center, large-scale, cross-sectional study; the cost of detection technology is high for early HCC screening in cohorts at high risk.	Auxiliary diagnosis	Chen et al. (2024)
——	——	Loss of large genomic regions in 5q and 16q arms	ultra-low-pass WGS	Patients with HCC (n = 73).	The findings of this study may be useful for prognosis evaluation and postoperative monitoring of HCC.	Auxiliary diagnosis	Sogbe et al. (2024)
——	——	550 cancer-specific regions	Target bisulfite sequencing.	Patients with HCC (n = 21) and benign liver masses (n = 4).	Longitudinal study revealed the relationship between methylation and tumor burden.	Prognosis assessment	Angeli-Pahim et al. (2024)
——	——	Read from No End-repair Enzymatic Methyl-seq (NEEM-seq)	NEEM-seq and a neural network (DeepTrace)	Patients with HCC (n = 62) and liver disease (n = 67), and healthy control (n = 39).	The stability of the methodology needs to be verified by independent cohorts and multi-centers.	Auxiliary diagnosis	Deng et al. (2023)
——	——	129 genes panel	target sequencing	Patients with HCC (n = 51)	The gene mutations of HCC are highly heterogeneous.	Auxiliary diagnosis and prognosis assessment	Cowzer et al. (2023)
GAMAD	NCT05626985	Gender, Age, Methylation, AFP, and DCP	chemiluminescence immunoassay and qMSP	Patients with HCC (n = 600), hepatitis or cirrhosis (n = 1,200), and healthy control (n = 200)	The methylation sites tested were not disclosed.		Yang et al. (2023)
I-score	——	CNV	NGS	patients with HCC (n = 37)	Sample size is small.	Prognosis assessment	Kim D. Y.et al. (2023)
MVFC	——	549 genes	target sequencing (NGS)	patients with HCC (n = 85)	This assay could be utilized in HCC tumor-burden monitoring and minimal residual disease detection.	Prognosis assessment and surveillance	Chen et al. (2023)
mt-HBT	NCT03628651	Model including three methylation markers (me*HOXA1*, me*TSPYL5*, and me*B3GALT6*), AFP, and gender.	qMSP	Control (n = 649) and patients with HCC (n = 292)	Enterprise-led studies with long duration and large sample size from multiple centers have a great hope of being translated into clinical application.	Auxiliary diagnosis	Chalasani et al. (2022), Singal et al. (2022)
——	——	*mSEPT9* in cfDNA and AFP, PIVKA-II	qMSP and immunoassays.	Patients with HCC (n = 154) and hepatic cirrhosis (n = 235)	Future large-scale screening studies are needed.	Auxiliary diagnosis	Zheng et al. (2023)

#### 5.2.1 Genomic level identification of cfDNA methylation markers

In the study of clinical application of liquid biopsy (Zeng et al., 2019), cfDNA methylation has been studied most extensively (Feng et al., 2019; Guo et al., 2017; Kang et al., 2017; Leygo et al., 2017; Xu et al., 2017; Zeng et al., 2018). In HCC, the 5-mc biomarkers of ctDNA has shown better diagnostic (such as serum AFP) and prognostic value (such as TNM staging) than current clinical indicators (Xu et al., 2017). In addition, repetitive sequences, such as long interspersed nuclear elements (LINE-1) and Alu repeats, are proxies for genomic methylation (Barchitta et al., 2014). In diffuse large B-cell lymphoma, DNA methylation of free LINE-1 shows a strong correlation with clinical outcomes, indicating its potential application as a prognostic marker (Wedge et al., 2017). Another approach to finding 5 mc markers is to identify tumor-specific methylated haplotypes as cell-free DNA markers to assess tumor burden and tumor origin sites (Guo et al., 2017). Multiple CpG haplotypes are more effective in tumor typing than single CpG markers (Guo et al., 2017). Recently, other types of epigenetic signatures, such as 5-hydroxymethylation (5-hmc) of cfDNA and nucleosome positioning and proportion, have also been used to assess tumor location and tumor (Chun-Xiao et al., 2017; Ivanov et al., 2015; Li et al., 2017; Snyder et al., 2016). Although the genome wide distribution of nucleosomes in cfDNA provides valuable information to identify the organizational origin of chaos in cfDNA, its clinical utility has been understudied.

Shen et al. (2018) from the University Health Network in Toronto, Canada, reported a study using MeDIP-Seq technology combined with machine learning to analyze the tumor methylation pattern of cfDNA. The investigators compared 300 patient tumor samples from seven disease sites (lung, pancreatic, colorectal, breast, leukemia, bladder, and kidney) with samples from healthy donors and analyzed methylation patterns in circulating cfDNA in plasma to track cancer origin and type. Although this study did not include liver cancer, it shows the potential of cfMeDIP-Seq for early cancer detection and localization. Nassiri et al. (2020) used cfMeDIP-seq technology to identify cfDNA methylation markers of intracranial tumors. Using cfMeDIP-seq, differentially hemi-methylated regions could serve as independent diagnostic biomarkers for HCC (Hua et al., 2024).

#### 5.2.2 Specific methylated genes or loci

Wong et al. (2000) and Wong et al. (1999) used Methylation specific PCR (MSP) to show that abnormal methylation of *CDKN2A* and *CDKN2B* genes, which encode p16 and p15 proteins, could be easily detected in the blood of HCC patients. The hypermethylation analysis of *RASSF1A* (ras association domain family 1A) in HCC patients showed that the sensitivity was 92.5% (37/40) in tissue and 42.5% (17/40) in plasma. *RASSF1A* hypermethylation was not detected in tissue samples, nor in plasma DNA samples (Yeo et al., 2005). Feng et al. (2010) used fluorescence quantitative PCR method (Methylight) to detect the methylation levels of 10 genes in liver tissues with 65 different viral infection states and found that the methylation levels of 5 genes such as *ACP*, *CDKN2A*, *HOXA9*, *RASSF1* and *RUNX* were significantly increased in malignant liver tissues. The methylation levels of *HOXA9*, *RASSF1* and *SFRP1* were higher in HBV-positive HCC samples. In contrast, *CDKN2A* was more methylated in HCV-positive HCC samples. The researchers used MSP to analyze 24 tumor suppressor genes and 3 cancer methylation sites in HCC tissues and adjacent tissues as well as chronic hepatitis tissues, and found that 8 tumor suppressor genes, including *HIC1*, *GSTP1*, *SOCS1*, *RASSF1*, *CDKN2A*, *APC*, *RUNX3* and *PRDM2*, were differentially expressed. Methylation levels are significantly elevated in early-stage HCC tissues and are associated with HCC progression in chronic hepatitis C. Therefore, it is speculated that the methylation changes of these genes play an important role in the early stage of cancer and are potential biomarkers for assessing the risk of chronic hepatitis C carcinogenesis (Nishida et al., 2012). Researchers also found that the methylation levels of different *TSGS* changed differently with the progression of HCC. The methylation levels of some tumor suppressor genes, including *RASSF2*, *MINT1*, *MINT2*, *RPRAM*, *SFRP2*, *CDH1* and *DCC*, changed more in the early stage of HCC than in the advanced stage. However, *CASP8*, *MINT31*, *PTGS2* and *CACNA1G* are the genes with methylation changes mainly in the high stage of liver cancer (Nishida et al., 2012). Zhou et al. (2018) conducted a meta-analysis of *CDKN2A* gene promoter region methylation and HCC risk. The results showed that the *CDKN2A* gene promoter region was hypermethylated in HCC, and its methylation level was significantly positively correlated with the risk of HCC (OR, 7.07; 95% CI, 5.67–8.80). In addition, this methylation is also positively correlated with HBV and HCV infection, the degree of cirrhosis, and age, which has the possibility of being HCC biomarker (Zhou et al., 2018). A study using bisulfite sequencing technology to analyze HCV-related liver tissues found that the promoter regions of Wnt suppressor genes *SFRP2* and *DKK1* were hypermethylated in the precancerous stages of chronic HCV infection, leading to Wnt silencing and eventual HCC development. The promoter region of *SFRP2* has the highest methylation level in HCC tissues, followed by cirrhosis tissues and chronic hepatitis tissues, and the lowest methylation level in normal liver tissues (Umer et al., 2014). Using methylation chip and MSP technology, researchers found 24 abnormally methylated CpG promoter regions, among which 4 hypermethylated genes (*TNFRSF10C*, *HOXA9*, *NPY* and *IRF5*) were downregulated in tumor tissues (Shin et al., 2010). Hernandez-Vargas et al. (2010) also used the same technical method to identify the unique methylation characteristics of HCC, which can distinguish HCC tumor tissues from paracancerous tissues and other types of tumor tissues. Furthermore, these specific methylation signatures are independent risk factors for cancer progression. Using Illumina Human Methylation 27K chip, Ammerpohl et al. (2012) detected and analyzed HCC (*N* = 12), cirrhosis (*N* = 15) and normal liver tissues (*N* = 12), and found that compared with normal liver tissues, A total of 167 hypomethylation sites and 100 hypermethylation sites associated with liver cirrhosis maintained a similar methylation pattern in HCC. Further analysis of the distribution of methylation levels in functional regions (promoter structures, PRC2-binding domains and CpG islands) across the genome revealed that the methylation patterns of cirrhosis and HCC were significantly different. A 450K methylation chip analysis was performed on 800 HCC patients, and 90% of HCC tissues showed hypomethylation or CpG island methylation phenotype at the genome-wide level. Similarly, another study found that most of the significantly hypermethylated CpG sites (60.1%) in liver cancer tissues were located on CpG islands, while the significantly hypomethylated CpG sites located on CpG islands accounted for only 8.2% of all hypomethylated CpG sites (SHI et al., 2016). Guo et al. (2017) performed genomic methylation analysis on ∼800 HCC samples, and selected 10 sites from 80 hypermethylated promoter sites as biomarkers of tumor and non-tumor tissues, with sensitivity and specificity close to 100%.

The scientists investigated the application of aberrant methylation of cfDNA in peripheral blood for the diagnosis of liver cancer. Zhang et al. (2007) used MSP technology to detect the methylation of *P15*, *P16* and *RASSF1A* in serum samples collected repeatedly before the diagnosis of liver cancer patients and normal people. The results showed that *RASSF1A* hypermethylation was detected at least once in 35 (70%) of 50 HCC patients, while p15 and p16 hypermethylation were detected in 22 (44%) and 12 (22%) of 50 HCC patients, respectively. The sensitivity and specificity of serum DNA methylation of these three genes as early predictive markers of HCC were 84% and 94%, respectively. SHI et al. (2016) analyzed the methylation differences between HCC patients’ cancer and adjacent tissues by methylation chip, and screened 2,324 differentially methylated CpG sites, including 684 hypermethylated sites and 1,640 hypomethylated sites in cancer tissues. Bisulfite Genomic Sequence (BSP) was used to detect the methylation levels of five genes (*CDKL2*, *STEAP4*, *HIST1H3G*, *CDKN2A* and *ZNF154*) in plasma samples from 38 patients. The feasibility of detecting plasma DNA methylation levels was confirmed. Wen et al. (2015) used MCTA-Seq to detect the cfDNA of 151 liver cancer patients and normal human controls, and found dozens of loci that could identify patients with early liver cancer. A classification model for plasma DNA identification of HCC patients achieved 94% sensitivity and 89% specificity. Wu et al. (2017) also used BSP to detect the methylation level of six genes *(CDKN2A*, *RASSF1A*, *STEAP4*, *TBX2*, *VIM* and *ZNF154*) in plasma DNA. The results showed that *TBX2* hypermethylation in plasma DNA was associated with an increased risk of HCC with an average odds ratios (ORs) of 3.2 (95% CI). Zhao et al. (2024) identified two novel methylation biomarkers, *GNB4* and *Riplet* through a genome-wide discovery, and showed sensitivity of 84.39% for any-stage HCC detection in plasma samples. Ruihua Xu from Sun Yat-sen University and Kang Zhang from University of California San Diego used Bisulfite padlock probes (BSPP) to detect cfDNA methylation. Ten early diagnosis and therapeutic related sites and eight prognostic related sites were identified from more than 400,000 candidate sites. The methylation levels of these ten early diagnosis sites showed a diagnostic sensitivity of 84.8% and a specificity of 93.1% in a total of 1098 HCC patients and 835 healthy controls. It can also accurately predict tumor staging, efficacy and recurrence (Xu et al., 2017). Methylation of *RNF135* and *LDHB* in cfDNA could be detected by Methylation Sensitive High-Resolution Analysis (MS-HRM) and could be used for HCC liquid biopsy (Kim D. Y. et al., 2023).

### 5.3 5-hydroxymethylcytosine (5 hmC) biomarkers

As one of the most important DNA modifications discovered in recent years, 5-hydroxymethylcytosine (5 hmC) is currently considered to be closely related to gene regulation and tumor pathogenesis, and can be used as a marker for early diagnosis of tumors. Unlike 5-methylcytosine (5 mC), which inhibits gene expression, 5 hmC is widely regarded as a marker of activated gene expression.

Li et al. (2016) used oxBS-Seq (oxidative bisulfite sequencing) to obtain methylation and hydroxymethylation profiles with single base resolution in the genome, and found that the enrichment characteristics of 5 hmc modification were tumor and tissue specific.

Starting with chemical marker detection technology, Professor He developed 5 hmc-seal technology with high sensitivity and stability, which can stably reproduce the sample detection under the requirements of trace DNA. Li et al. (2017) studied the molecular characteristics of 5 hmc in 90 healthy people, 260 cancer patients (including 80 colorectal cancer, 71 gastric cancer, 25 liver cancer, 34 pancreatic cancer, 46 thyroid cancer) and 71 benign gastrointestinal diseases. 5 hmC sequences in tissue and peripheral blood cfDNA were captured by 5 hmc-SEAL technology, and 5 hmC modification levels were analyzed by high-throughput sequencing technology. After normalization of 5 hmc modification levels, bioinformatics tools were used to find 5 hmC gene loci with significant differences in modification levels between the patient group and the normal group. Subsequently, the regression model was used to further screen and optimize the difference sites obtained as above, so as to obtain a set of 5 hmC biomarkers with high sensitivity and specificity. The obtained biomarkers were further validated in independent samples. This study reveals the potential of 5 hmc as a molecular marker for clinical application, especially in early cancer screening and cancer localization and classification.

At the same time, it was also explored that the value of cfDNA 5 hmC for tumor diagnosis (Chun-Xiao et al., 2017). A total of 49 patients with primary tumors were included in the study, including 15 lung cancer, 10 HCC, 7 pancreatic cancer, 4 glioma, 5 gastric cancer, 4 colorectal cancer and 4 breast cancer samples. It was found that tSNE dimensionality reduction analysis of the enrichment degree of all genes 5 hmC could distinguish HBV infection, HCC and healthy human samples. HCC-specific hypermethylated and hypomethylated genes can be used to distinguish HCC, some HBV samples and healthy samples. The high hydroxymethylated genes characteristic of HCC are also highly expressed in liver tissue, which is also consistent with the permissive effect of 5 hmC on gene expression. Subsequently, Stephen R Quake and Dan Xie’s team also collected blood samples from 4 HCC patients after surgery, as well as samples from 3 recurrent HCC patients, and found that the postoperative samples returned to the level of healthy samples, while the changes of markers in recurrent HCC samples were similar to those in HCC samples. Similarly, the change trend of 5 hmC enriched gene *AHSG* and lacking gene *TET2* can also be used to observe the change of 5 hmC in the short term after surgery until recurrence. These results suggest that cfDNA 5 hmC sequencing technology has the potential to be used in the detection, diagnosis, treatment and recurrence monitoring of liver cancer.

Jia Fan from Zhongshan Hospital, Fudan University and Hongyang Wang from National Liver Cancer Science Center/Eastern Hepatobiliary Surgery Hospital, together with Chuan He from the University of Chicago and Wei Zhang from Northwestern University, further studied the application value of peripheral blood 5 hmC technology in the early diagnosis and screening of liver cancer. Genome-wide mapping of 5 hmc in cell-free DNA samples was performed in 2,544 subjects (1,204 with HCC, 392 with chronic hepatitis B or cirrhosis, 958 with healthy or benign liver damage). A case-control analysis using elastic network regularization for feature selection developed a model using 32 genes for early HCC diagnosis. The results showed that the 5 hmC model not only accurately distinguished patients with early-stage liver cancer from healthy controls, but also could distinguish patients with early-stage liver cancer from patients with chronic hepatitis B or cirrhosis, and its prediction performance was significantly better than that of AFP. However, combining 5 hmC markers with AFP can further improve the performance of AUC (Cai et al., 2019). Liu et al. (2019) found that in the early stage of HCC, 5 hmc and 5 fmc modification levels were significantly reduced, mainly due to the reduction of 5 mc modification levels, and were related to HBV infection, reduced TET enzyme activity, and abnormal expression of DNA methylation-related enzymes.

Hlady et al. (2019) integrated and analyzed the genomic epigenetic modifications of liver cancer, cirrhosis and normal liver tissues, including the histone modifications of 5 mc, 5 hmc and the promoter/enhancer regions of four genes (*H3K4me1*, *H3K27ac*, *H3K3me3* and *H3K27me3*). The results demonstrated that these modifications jointly regulated gene expression and subsequently affected the proliferation of HCC cells. By integrated analysis of epigenetic modifications, two epigenetic driver sites of HCC, *COMT* and *FMO3*, were also identified.

### 5.4 Markers of somatic mutation

The initiation and development of tumors are essentially caused by the activation of proto-oncogenes and the inactivation of tumor suppressor genes. For example, the Ser249 site of tumor suppressor gene *TP53* is the most reported hotspot mutation in HCC (Szymańska et al., 2004). Mutations at this site cause the tp53 protein to lose its ability to bind specifically to DNA. It has been reported that *TP53* Ser249 mutation in plasma DNA is associated with cirrhosis and HCC (Hosny et al., 2008; Kirk et al., 2005). This mutation was also detected in paracancerous tissues of HCC patients, suggesting that it may be involved in the early development of HCC and accumulated during the progression of HCC (Aguilar et al., 1994). Like many other point mutations, the Ser249 variant in *TP53* has been found in samples from other cancer types. In recent years, cancer genome studies have found that most HBV-related HCC patients have at least one mutation in *TP53*, *CTNNB1*, *AXIN1* and *TERT* promoter, and most of these mutations appear in the early stage of liver cancer, showing their close relationship with liver cancer development (Totoki et al., 2014; Zhang et al., 2017). Other somatic mutations that may be involved in the occurrence of liver cancer include *ARID1A*, *ARID2*, *NFE2L2*, *KEAP1*, *JAK1* and *RPS6KA3* (Guichard et al., 2012; Kan et al., 2013; Nault et al., 2014; Schulze et al., 2015; Totoki et al., 2014).

### 5.5 Other molecular features of cfDNA serve as markers

Professor Lo of the Chinese University of Hong Kong has led a team of researchers to discover the DNA in the plasma of patients with HCC by massively parallel sequencing technology. The results confirmed the existence of DNA fragments with abnormal lengths in the plasma of HCC patients. The shorter DNA molecules are more likely to carry some tumor-associated copy number abnormalities. It has also been found that the amount of mitochondrial DNA in the plasma of HCC patients is increased. These mtDNA molecules are shorter than plasma nuclear derived DNA (Jiang et al., 2015). In another study, the same team found that ccfDNA with breakpoints at specific locations in the genome was most likely derived from liver cancer cells. This finding reveals the possibility that plasma DNA breakpoint signatures can be used as biomarkers for liquid biopsy that are easier to detect than somatic mutation markers (Jiang et al., 2018). Wen et al. (2015) found that the concentration of cfDNA in liver cancer patients varies greatly and is independent of tumor size. Yan et al. (2018) found that the total amount of cfDNA in HCC patients was significantly higher than that in non-patients. With the constructed HCC diagnostic model including age, cfDNA content, and AFP, 87% sensitivity and 100% specificity could be achieved. In recently, fragmentomics patterns of cfDNA were investigated and provides new insights into the biological properties and potential clinical applications of noninvasive detection of HCC (An et al., 2023; Che et al., 2024; Deng et al., 2023; Gonçalves et al., 2022; Liu et al., 2023; Xu et al., 2024).

### 5.6 Small RNA (miRNA) markers

MiRNAs are a class of small single-stranded RNAs of about 22 nt that are processed from transcripts of hairpin structures produced endogenously in cells. Chen et al. (2008) from Nanjing University found that a large number of miRNAs stably exist in serum and plasma, which constitute the main part of circulating nucleic acids in serum. These miRNAs have good tolerance to different temperatures, pH values, storage time, repeated freezing and thawing, etc., which may be related to the fact that miRNA is mostly bound to proteins and encapsulated in exosomes and protected (Cheng et al., 2014).

In recent years, numbers of studies have confirmed that the detection of miRNA expression levels in tissues and serum can be used for the diagnosis, prediction of pathological classification and prognosis of liver cancer, and has certain reference value in the screening and follow-up of liver cancer. Zhou et al. (2011) used chip technology to screen 723 miRNAs in 934 HBV-positive liver cancer patients, and designed a diagnostic panel containing 7 miRNAs, with a sensitivity and specificity of 68.6% and 90.1%, respectively. The accuracy of this method in distinguishing HCC from normal subjects, hepatitis from cirrhosis was 94.1%, 84.2% and 88.4%, respectively. An *in vitro* diagnostic kit for this method has been developed and approved by National Medical Products Administration (NMPA). Studies on exosomal miRNA expression levels found that miR-18a, miR-221, miR-222 and miR-224 were significantly increased in the serum of HBV-infected HCC patients (Hayes and Chayama, 2016; Sohn et al., 2015). Jin et al. (2019) also found 12 plasma miRNA markers highly expressed in HCC patients from more than 700 miRNAs.

### 5.7 Combined detection of multiple types of markers

Cohen et al. (2018) combined the results of somatic mutation detection based on single-molecule tags and protein markers (CA125, CEA, cancer antigen 19–9, prolactin, hepatocyte growth factor, etc.) in 8, and constructed the CancerSEEK model by comparing cancer patients and healthy controls. The results showed that 43 of 44 (98%) HCC patients were detectable. HCCscreen, which detects four point mutations and one HBV integration site in cfDNA in combination with two protein markers, was developed by several scientists from National Cancer Center/Cancer Hospital, Chinese Academy of Medical Sciences. It was named HCCscreen and its application in the early screening of liver cancer in a prospective cohort of HBV carriers. In this study, 331 asymptomatic HBV carriers with normal AFP and B ultrasound were screened and followed up for 6–8 months. The results showed that among the 24 positive samples, 4 cases were subsequently diagnosed as liver cancer. Due to the early detection, 4 patients were expected to get a good prognosis. None of the 307 participants who tested negative developed liver cancer. In this study, the HCCscreen combined detection scheme achieved 100% sensitivity, 94% specificity and 17% positive predictive value, which is expected to be applied to early screening of liver cancer (Qu et al., 2019). By DNA sequencer-assisted fluorophore-assisted carbohydrate electrophoresis, the blood glucose spectrum of patients with HCC were evaluated, and the log ratio of peak 9 to peak 7 was named the GlycoHCCTest (G-test). The G-test is a promising noninvasive method to screening HCC in patients with chronic hepatitis B and cirrhosis (de Oliveira et al., 2018; Wan et al., 2021), and obtained the NMPA Class III medical device registration certificate for non-invasive detection of HCC (Cong et al., 2020; DelaCourt et al., 2021). Integrating cfDNA methylation, copy number, and fragmentation using 950 plasma and 240 tissue samples, which could to facilitates multi-cancer (including HCC) early detection (Kim S. Y. et al., 2023). CAMP-B score (cirrhosis, age ≥50 years, male sex and platelet count <150,000/mm3/L) can be easily implemented in real-world clinical practice and helps stratify HCC risk in patients with CHB following HBsAg seroclearance (Lee et al., 2024). Its application in HCC screening needs further investigate.

## 6 Summary

Noninvasive detection of liver cancer is very important for improving the prognosis and quality of life of patients. There are still many shortcomings in the current screening methods in clinical application, and there is an urgent need for new assays that are more reliable, convenient and less expensive. Future research prospects focus on two aspects: a. Optimization and combined application of traditional markers; b. Development of novel blood markers.

DNA methylation has advantages as a biomarker and has been clinically applied in a variety of cancers. The advantages of DNA methylation over other markers such as proteins and RNA include: a. Higher Specificity: DNA methylation patterns can provide more specific information related to disease states compared to protein markers, which may have broader implications across various conditions. Stability: DNA methylation patterns are relatively stable in clinical samples, making them robust biomarkers for long-term storage and analysis compared to RNA, which can degrade more easily. Early Detection Potential: Changes in DNA methylation can occur early in disease progression, offering potential for early detection of conditions like hepatocellular carcinoma (HCC) before clinical symptoms manifest. Quantifiability: DNA methylation levels can be quantitatively measured with high precision, allowing for accurate monitoring of disease progression and treatment efficacy. Technological Accessibility: Advances in technology have made DNA methylation analysis increasingly accessible and cost-effective, facilitating its integration into clinical diagnostics. These advantages collectively highlight DNA methylation as a promising biomarker for improving diagnostic accuracy and clinical outcomes in diseases like HCC. There are many studies on the application of DNA methylation in the early screening, diagnosis, and postoperative monitoring of liver cancer, but there is still a lack of methylation markers that can be applied to clinical promotion. Finding sensitive and specific DNA methylation markers of liver cancer at the genomic level may be a feasible research scheme to realize its clinical application in early screening and early diagnosis.

## Abbreviations

HCC, hepatocellular carcinoma; HBV, hepatitis B virus; HCV, hepatitis C virus; HEV, hepatitis E virus; MFLD, Metabolic fatty liver disease; AFP, alpha-fetoprotein; cfDNA, cell-free DNA; DMR, differentially methylated regions; NGS, Next-generation sequencing; qMSP, quantitative methylation-specific polymerase chain reaction; PBMC, peripheral blood mononuclear cell; PCR, polymerase chain reaction.
